# Supplementary material for: mHealth technology for ecological momentary assessment in physical activity research: a systematic review
Source: PeerJ. 2020 Mar 26;8:e8848. doi: 10.7717/peerj.8848 (PMC7103204; doi:10.7717/peerj.8848)
Supplement: Supplemental Information 2 [file peerj-08-8848-s002.pdf]

Supplementary File: Search strings for all databases in the systematic review.

Table 1. Search strings for PsycINFO (by PsycNET)

1	"Ecological momentary assessment" OR "experience sampling" OR "experience samplings"
2	Exercise OR Exercises OR “Physical activity” OR “physical activities” [APA Thesaurus]
3	#1 AND #2
4	Methodology: Clinical Case Study OR Clinical Trial OR Empirical Study OR Experimental Replication OR Follow up Study OR Longitudinal Study OR Prospective Study OR Retrospective Study OR Field Study OR Nonclinical Case Study OR Qualitative Study OR Quantitative Study OR Twin Study
5	FILTER: 2008 to 2018
6	FILTER: Population Group: humans

Table 2. Search strings for CINAHL

1	"Ecological momentary assessment" OR "experience sampling" OR "experience samplings"
2	Exercise OR Exercises OR “Physical activity” OR “physical activities”
3	#1 AND #2
4	FILTER: Human
5	FILTER: 2008 to Feb/2018

Table 3. Search strings for Medline (by Pubmed)

1	"Ecological momentary assessment" OR "experience sampling" OR "experience samplings"
2	Exercise OR Exercises OR “Physical activity” OR “physical activities”
3	#1 AND #2
4	All fields: Articles type: case reports, classical article, clinical study, clinical trial, clinical trial phase I, clinical trial phase II, clinical trial phase III, clinical trial phase IV, comparative study, controlled clinical trial, journal article, twin study
5	FILTER: Publication dates: 2008 to 13/FEB/2018
6	FILTER: Species: humans

Table 4. Search strings for Web of Science core collection (by Web of Science)

1	"Ecological momentary assessment" OR "experience sampling" OR "experience samplings"
2	Exercise OR Exercises OR “Physical activity” OR “physical activities”
3	#1 AND #2
4	Refined by: document types: (article or proceedings paper) and [excluding] categories of web of science: (engineering electrical electronic and anthropology and biotechnology applied microbiology and computer science hardware architecture and computer science software engineering and computer science theory methods and mathematical computational biology and computer science artificial intelligence and rheumatology and computer science information systems and urology nephrology and veterinary sciences and information science library science)
5	FILTER: 2008 to 2018
6	Citation Indexes: SCI-EXPANDED, SSCI, A&HCI, CPCI-S, CPCI-SSH, BKCI-S, BKCI-SSH, ESCI, CCR-EXPANDED, IC
