# Supplementary material for: mHealth technology for ecological momentary assessment in physical activity research: a systematic review
Source: PeerJ. 2020 Mar 26;8:e8848. doi: 10.7717/peerj.8848 (PMC7103204; doi:10.7717/peerj.8848)
Supplement: Supplemental Information 3 [file peerj-08-8848-s003.pdf]

Our work is unique to the present time by making a systematic review about the ability of mHealth technology to apply Ecological Momentary Assessment (EMA) methodology in the field of physical activity (PA) behavior. We have focused on the instruments and procedures of applying mHealth technology for monitoring PA behaviors in real time and in natural environments. We included data from the last decade, from the massive emergence of smartphones as multi-tasking tools capable of monitoring lifestyles of the population. We also synthesized the methodological and procedural aspects of EMA in all type of populations and suggested methodological recommendations regarding its use.

No review was published similar to the one we want to publish. Existing systematic reviews of EMA interventions focus on specific age groups like young people (see references 9 and 14); or about some aspect of the lifestyle other than the PA, such as emphasizing sedentary behavior (see reference 15); or are based on an excessively strict list of criteria that does not include all EMA studies according to their original definition (see references 14,15). This checklist becomes very demanding when you want to describe some published studies that, in general terms, comply with the basic features of the EMA methodology. For instance, in a recent systematic review using this checklist to assess sedentary behavior in articles published between 2007 and 2017, only 21 of 50 potential studies were included. It is surprising that only four of these works are combining EMA with some objective measurement like accelerometry (see reference 15).

We did not use this checklist because we were interested in identifying studies that have used EMA or similar basic procedures since the emergence of smartphones on the market. A total of 76 studies in 74 articles on the use of EMA and PA, in general population of all ages, were included for the qualitative synthesis. We have found enough evidence to firmly propose the use of the term mEMA (mobile-based EMA) when mHealth technology is being used for monitoring real-time lifestyle behaviors in natural situations.
